# Supplementary material for: Total Triterpenes, Polyphenols, Flavonoids, and Antioxidant Activity of Bioactive Phytochemicals of Centella asiatica by Different Extraction Techniques
Source: Foods. 2023 Oct 30;12(21):3972. doi: 10.3390/foods12213972 (PMC10647812; doi:10.3390/foods12213972)
Supplement: Supplementary file 1 [file foods-12-03972-s001.zip › Supplementary table ST1 .pdf]

Supplementary table ST1 CIE L*a*b* colour space measurements taken before and after air dying of *C. asiatica* leaf samples

	Before drying			After drying		
Value	L*	a*	b*	L*	a*	b*
1	39.69	+0.62	-1.05	40.60	+0.09	-0.01
2	38.30	+1.51	-2.29	39.11	+1.30	-1.72
3	41.82	+0.79	+0.64	40.12	+1.03	-1.31
Average	**39.93**	**+0.973**	**-0.90**	**39.94**	**+0.81**	**-1.01**

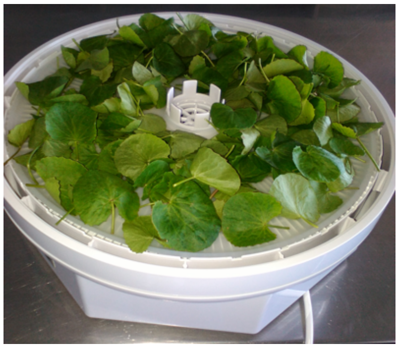

Fresh leaves

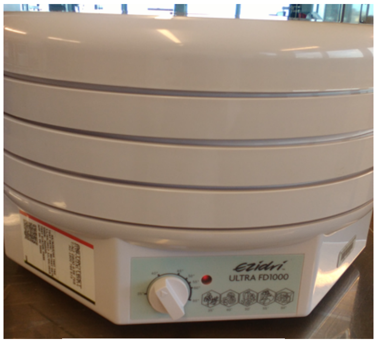

Air drying

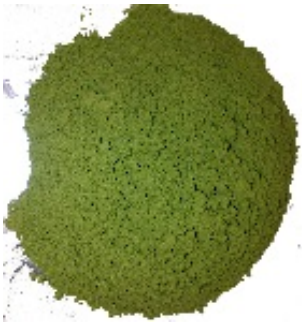

Dried powder
