# Supplementary figures and images for: Total Triterpenes, Polyphenols, Flavonoids, and Antioxidant Activity of Bioactive Phytochemicals of Centella asiatica by Different Extraction Techniques
Source: Foods. 2023 Oct 30;12(21):3972. doi: 10.3390/foods12213972 (PMC10647812; doi:10.3390/foods12213972)

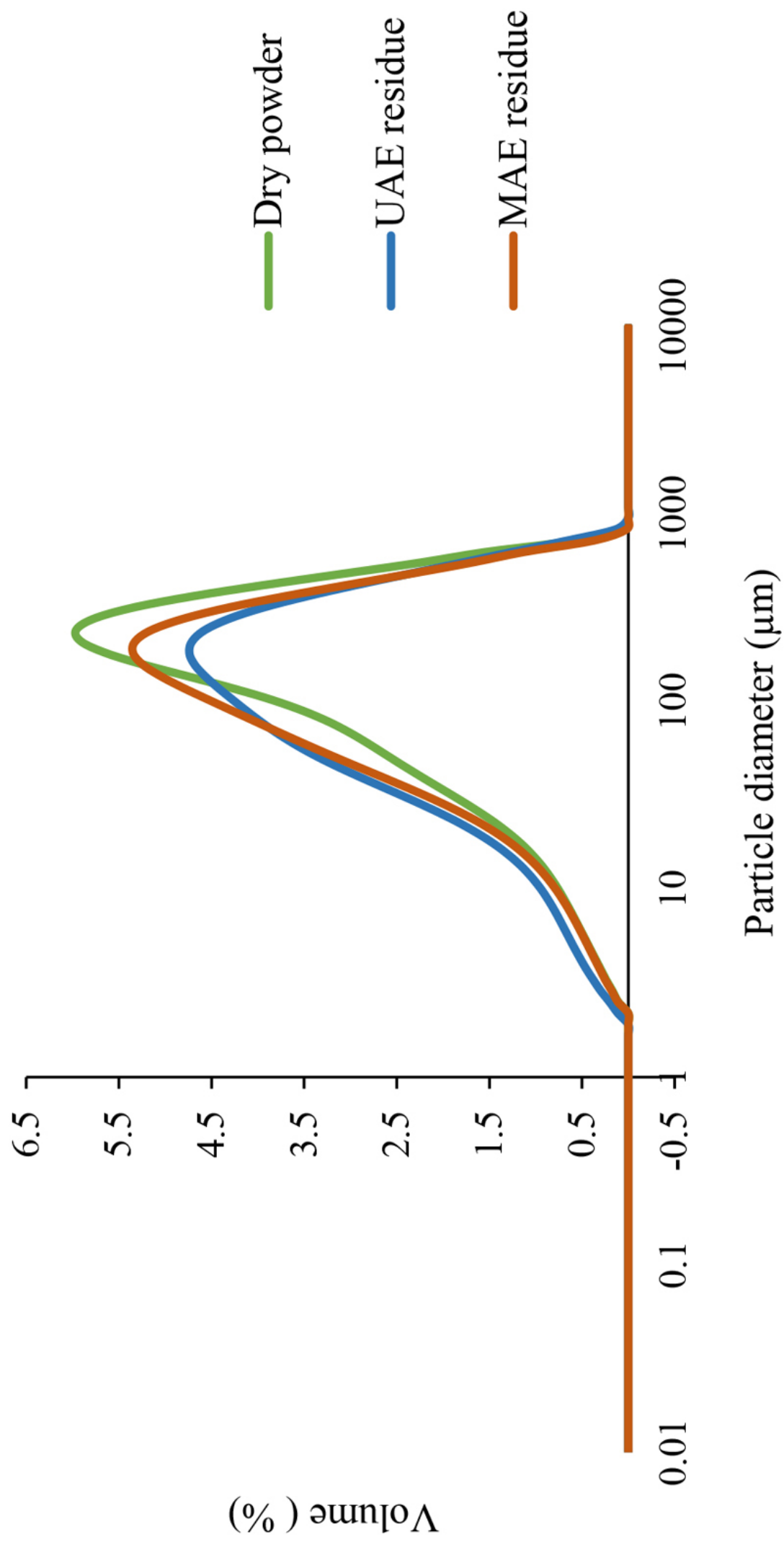

Supplement: Supplementary file 1 [file foods-12-03972-s001.zip › Supplementary Figure S1.pdf]

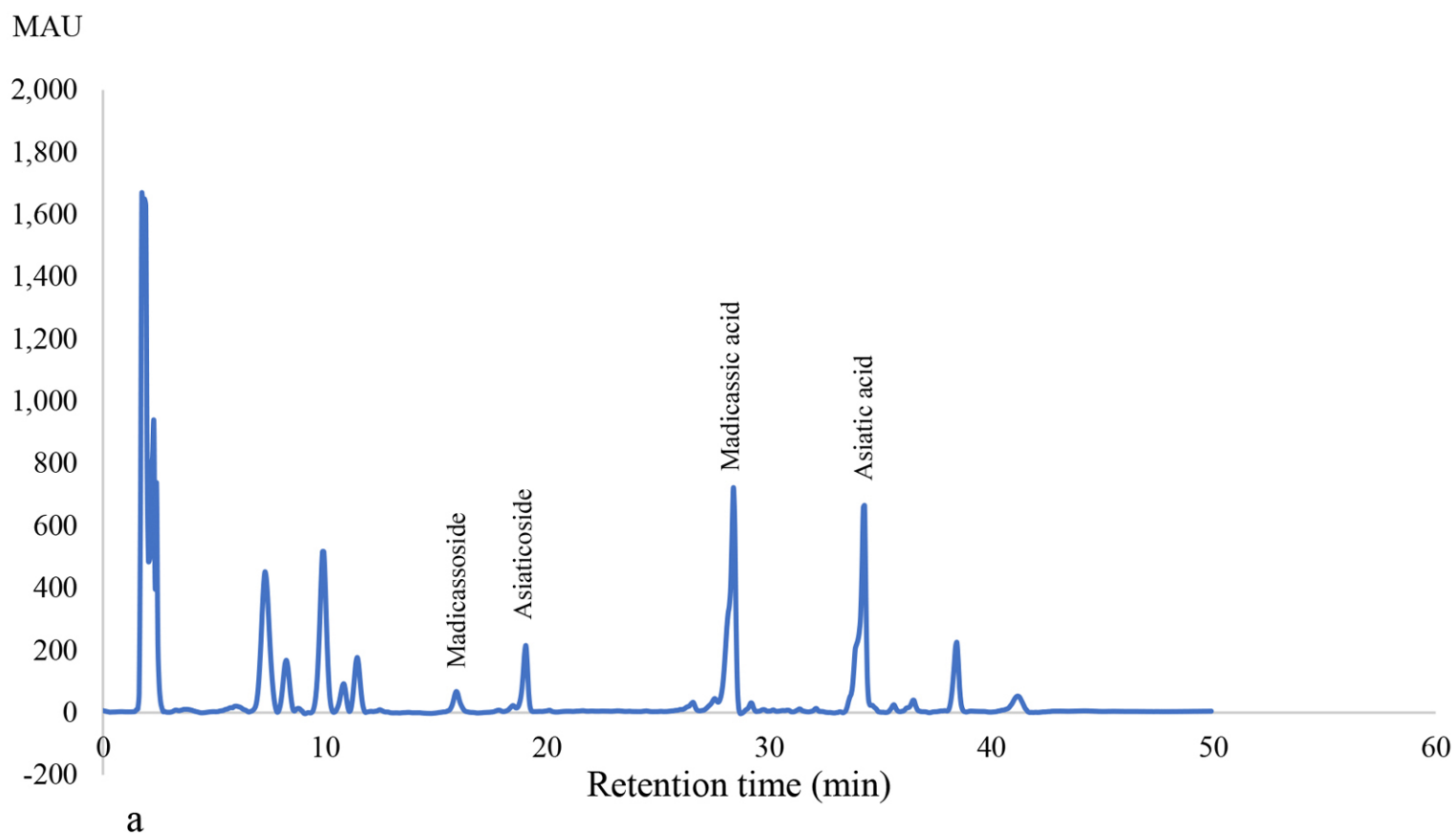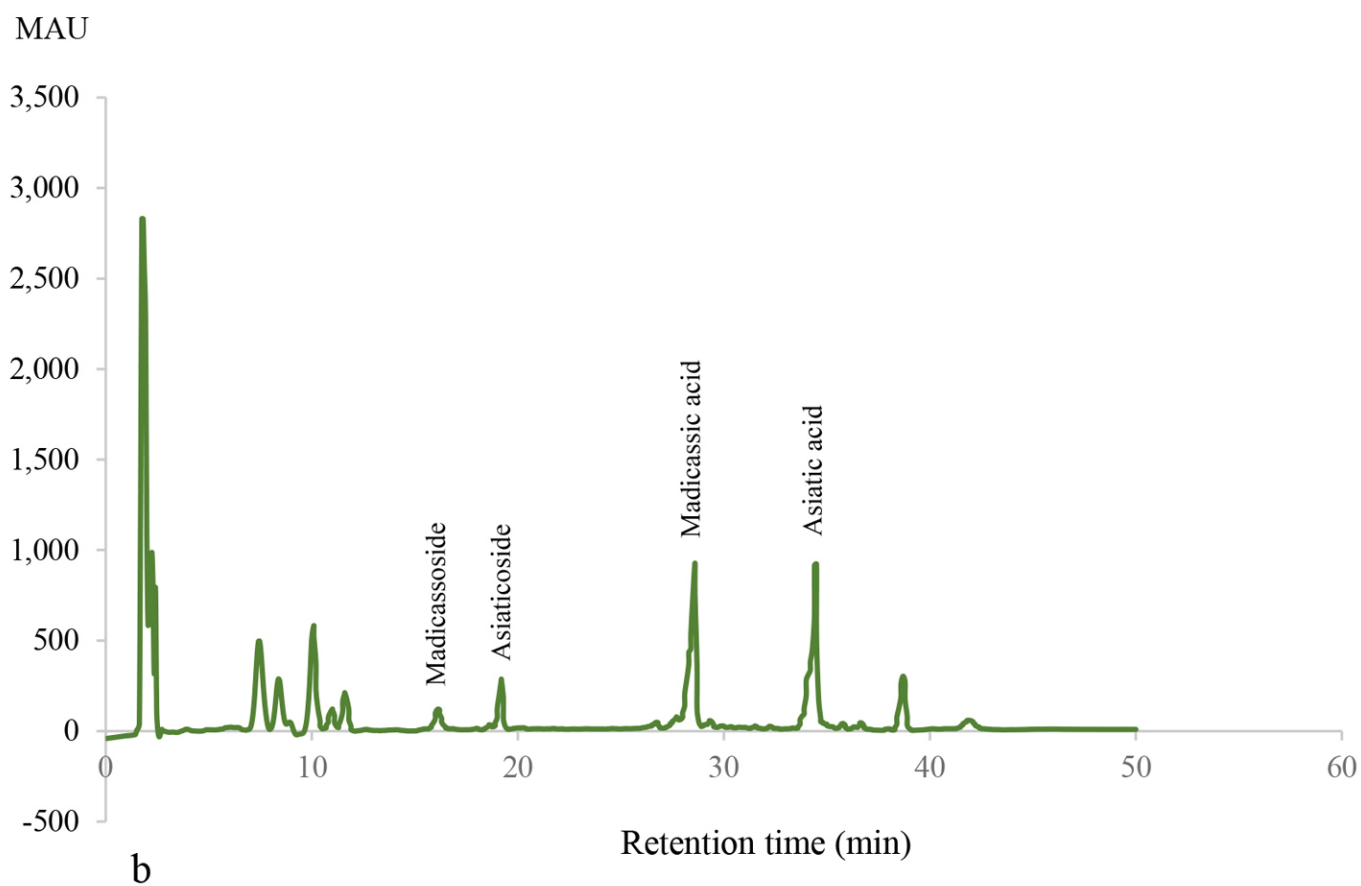

Supplement: Supplementary file 1 [file foods-12-03972-s001.zip › Supplementary Figure S2.pdf]
